# CCND3 Suppression Ameliorates β-Thalassaemia in a Murine Disease Model: A Potential Therapeutic Strategy

**DOI:** 10.3390/cells15060495

**Published:** 2026-03-10

**Authors:** Cristian Antonio Caria, Maria Franca Marongiu, Susanna Porcu, Daniela Poddie, Simona Vaccargiu, Jim Vadolas, Alessandra Meloni, Lucia Perseu, Alessandra Olianas, Maria Serafina Ristaldi

**Affiliations:** 1 Istituto di Ricerca Genetica e Biomedica, Cittadella Universitaria di Monserrato, SS 554, Bivio Sestu Km 4.500, 09042 Cagliari, Italy; cristianantonio.caria@cnr.it (C.A.C.); mariafranca.marongiu@cnr.it (M.F.M.); susanna.porcu@cnr.it (S.P.); daniela.poddie@cnr.it (D.P.); simona.vaccargiu@cnr.it (S.V.); alessandra.meloni@cnr.it (A.M.); lucia.perseu@cnr.it (L.P.); 2 Centre for Cancer Research, Hudson Institute of Medical Research, Melbourne 3168, Australia; jim.vadolas@hudson.org.au; 3 Department of Molecular and Translational Science, Monash University, Melbourne 3168, Australia; 4 Department of Life and Environmental Sciences, Statal University of Cagliari, 09042 Monserrato, Italy; olianas@unica.it

**Keywords:** β-thalassaemia, drug therapy, erythropoiesis, HbA2, HbF

## Abstract

β-thalassaemia (β-thal) is part of a group of diseases, the β-hemoglobinopathies, affecting the levels or functionality of the β-globin subunit of hemoglobin, which are the most widespread monogenic diseases throughout the world. The severity of β-thal is determined by different genetic factors, but in the gravest form, affected patients are constrained to a program of blood transfusion and iron chelation regimens for their entire life. Although definitive cures, such as bone marrow transplantation or gene therapy, are now available, they are still far from being applied worldwide. Therefore, there is growing attention towards the use of drugs to cure or ameliorate β-thal disorder. Among all the strategies, pharmacological increase of fetal HbF and/or adult HbA2 can represent an advantageous approach as high levels of both hemoglobins are effective against β-thal. Therefore, the identification of therapeutic targets that can modulate, by the use of drugs, these hemoglobins is increasingly urgent. In this paper, we analyze the effects of the absence of the *CCND3* gene, a druggable target associated with HbF and HbA2 levels, in a humanized mouse model of β-thal to assess the impact against the disorder. Upregulation of γ- and δ-globin levels in mice lacking *Ccnd3* expression contributes to partial restoration of the α/β balance, with a consequent increase in hemoglobin levels, improvement of iron levels, and reduction of splenomegaly. Moreover, we present data supporting the enhancement of erythropoiesis. Our data indicate the *CCND3* gene as a possible target for drugs against β-thal.

## 1. Introduction

β-thalassaemia (β-thal; MIM: 613985) is a monogenic disease belonging to the heterogeneous group of β-hemoglobinopathies, which are the most common monogenic diseases worldwide [1,2,3,4,5]. In β-thal, mutations cause a partial or total absence of β-globin, leading to an accumulation of unbound α-globin chains in differentiating erythroblasts. These excess α-chains form unstable tetramers that precipitate, triggering oxidative damage and ultimately leading to anaemia and ineffective erythropoiesis (IE) [6]. Depending on its severity, β-thal is divided into three categories: β-thal minor (β-thal carriers), transfusion-dependent thalassaemia (TDT, formerly thalassaemia major), and non-transfusion-dependent thalassaemia (NTDT, formerly thalassaemia intermedia) [7]. The minor form is the mildest and is often almost asymptomatic. In contrast, untreated TDT is defined by severe microcytic anaemia (Hb < 7 g/dL) and characteristic alterations in erythrocyte morphology, along with a clinical picture including growth retardation, hepatosplenomegaly, and skeletal abnormalities as a consequence of massive erythropoietic expansion [1]. Treatment of TDT involves a lifelong regimen of blood transfusion. However, while this therapy leads to the improvement of pathological symptoms by maintaining high hemoglobin (Hb) levels, it is accompanied by chronic iron overload, which leads to other complications such as endocrinopathies and cardiovascular disease [8,9], necessitating concomitant iron chelation therapy [1,10]. Moreover, blood transfusion regimens can lead to immune involvement, which may subsequently result in haemolysis, as observed in delayed haemolytic transfusion reactions [11]. NTDT is a milder form of the disease, with the need for transfusion being rare or even absent. However, IE is present in patients affected by NTDT to varying degrees, as well as symptoms like hepatosplenomegaly, bone alterations, and iron overload, which is determined by a transfusion-independent mechanism [12,13,14,15].

So far, the definitive cures for β-thal are gene therapy and bone marrow transplantation. However, the effectiveness of bone marrow transplantation is limited by the patient’s age and clinical condition at the time of engraftment, as well as by HLA compatibility between donor and recipient [16,17]. As for gene therapy, while potentially curative, it is hampered by its high cost, which limits its global accessibility [18,19,20] and requires further long-term evaluation to fully ascertain its safety and efficacy [21,22]. Therefore, alternative therapies capable of curing or improving the symptoms of the disease are becoming increasingly necessary. In this context, the use of drugs may represent a pivotal strategy, given their potential for global distribution. Several approaches have been investigated that focus on different aspects of the disease. Among these, drugs like Luspatercept and Sotapercept aim to counteract IE directly by inhibiting the SMAD2/3 signalling pathway and, consequently, relieving the inhibition on late-stage erythroblast differentiation [23,24,25,26]. Another approach focuses on preventing iron overload through the stimulation of Hepcidin expression or pharmacologically targeting its downstream effectors [27]. Moreover, Mitapivat, recently approved by the FDA for α- and β-thal, is a small-molecule pyruvate kinase activator that increases ATP production in red blood cells (RBC). In patients, it raises Hb levels and improves symptoms such as iron overload and IE. It is well tolerated, with no major adverse effects, and is the first oral therapy for anaemia in both TDT and NTDT patients [28,29,30].

An additional key strategy is to rebalance the α/β-globin ratio by inducing the expression of β-like globin, such as fetal γ-globin and/or adult δ-globin. The therapeutic potential of elevating γ-globin is well established, especially since the discovery of natural mutations that free β-thal patients from lifelong transfusion regimens [31,32,33]. In this context, hydroxyurea represents a valuable and widely used drug in the treatment of sickle cell disease [MIM: 603903] and NTDT, although it has many limitations. Moreover, its efficacy in severe β-thal has not yet been established [27]. Notably, a recent work from Orkin’s group highlighted the possibility of pharmacologically blocking γ-globin silencing by avoiding tetramerization of BCL11 transcription factor A (BCLL11a), a known regulator of γ-globin expression [34,35,36,37,38], opening new possibilities for the treatment of β-hemoglobinopathies [39]. Another highly valuable target, whose efficacy in the treatment of β-hemoglobinopathies has been recently proven in murine models of the disease, is the δ-subunit of HbA2 adult Hb, which has the advantage, compared to γ-globin, of being pan-cellularly expressed [40,41,42,43]. Moreover, the possibility of modulating δ-globin levels by pharmacological treatment has been recently highlighted [41].

Recently, our lab ascertained the potential of the *cyclin D3* (*CCND3*, [MIM: 123834]) gene, previously associated with HbF and HbA2 levels by GWAS [44], in modulating g- and d-globin expression in a transgenic mouse model containing the entire human β-globin cluster [45,46]. Furthermore, we recently generated a murine cell line designed for the screening of active compounds in preclinical settings. In this system, we demonstrated that pharmacologically modulating Ccnd3 activity with a repurposed cancer drug leads to a promising elevation of both γ- and δ-globin levels [47]*. CCND3* is a key regulator of cell cycle progression, promoting the G1/S phase transition through its interaction with CDK4/6 and the retinoblastoma (pRB) pathway [48,49,50]. In humans, variants involved in *CCND3* modulation have been associated with haematological parameters such as RBC count, mean corpuscular volume (MCV), and mean corpuscular hemoglobin (MCH) [51,52,53,54,55]. Likewise, mice lacking *Ccnd3* expression show increased volumes but reduced erythrocyte counts [56].

In this study, we investigated the effects of *Ccnd3* deprivation in a humanized mouse model of β-thal that contains the complete human beta-globin gene cluster [57].

Our data show an overall improvement in the β-thal phenotype in mice lacking *Ccnd3* expression. This improvement is characterized by a significant increase in β-like globin expression, resulting in elevated Hb levels, improved iron levels, and partial recovery from IE. Overall, these data suggest that the *Ccnd3* gene could be a promising target for pharmacological therapy for β-thal.

## 2. Materials and Methods

### 2.1. Mice

The transgenic mouse line carrying the 4 bp deletion (referred to as Δ4bp) [57] was provided by the University of Queensland Biological Resources (Brisbane, Australia). The Hbbth3/+ mouse line [58] was obtained from the Memorial Sloan Kettering Cancer Center (New York, NY, USA). The *Ccnd3*−/− mouse line [56] was kindly provided by Dr. Piotr Sicinski (Dana-Farber Cancer Institute, Harvard Medical School, Boston, MA, USA). This study was conducted according to the Italian (D.Lgs 26/2014) and EU directives for animal experimentation (2010/63/EU, L.276; 22 September 2010), the guidelines issued by the Committee for Animal Welfare (OPBA) of the University of Cagliari and authorized by the Italian Ministry of Health (Aut n. 780/2021-PR). All the appropriate procedures were followed to minimize animal discomfort and the number of animals used.

For genotyping, genomic DNA was isolated from tail clips and genotyped by polymerase chain reaction (PCR) using standard protocols from The Jackson Laboratory (protocol access date: 8 March 2023). The sequences of all primers used for genotyping are listed in Table S1.

### 2.2. Real-Time PCR

Total RNA was extracted from the bone marrow of adult mice (age 4–8 months) using TRIzol LS reagent (Thermo Fisher Scientific, Boston, MA, USA), following the manufacturer’s instructions. Following DNase I treatment (Thermo Fisher Scientific, Boston, MA, USA), RNA was reverse-transcribed into cDNA using Superscript III reverse transcriptase (Thermo Fisher Scientific, Boston, MA, USA). RT-qPCR was performed using SYBR Green chemistry on an ABI PRISM 7900 thermocycler (both from Thermo Fisher Scientific, Boston, MA, USA). Each reaction was run in technical triplicate on a minimum of three independent biological samples. The expression of fetal and adult globin genes was quantified relative to mouse or human α-globin mRNA using the 2^−ΔΔCt^ method. Proportions of γ-, δ-, and β-globin transcripts were calculated from relative qPCR measurements. Ct values were normalized to mouse α-globin as endogenous reference (ΔCt = Ct_target − Ct_α) and converted to linear values using the 2^−ΔCt^ method. The relative contribution of each β-like globin was expressed as a fraction of the total β-like globin pool according to the formula: (2^−ΔCt^_x)/(2^−ΔCt^_γ + 2^−ΔCt^_δ + 2^−ΔCt^_β) and expressed as a percentage. Primer sequences are listed in Table S1.

### 2.3. Haematology

Blood samples (0.2 mL) were collected via cardiac puncture from euthanized adult mice into EDTA-coated Microtainer tubes. Haematological parameters, including Hb concentration, were measured using an automated hematology analyzer (MS4, Melet Schloesing Laboratories, Osny, France).

### 2.4. Flow Cytometry

Analyses were conducted on freshly isolated cells (1 × 10^5^ per sample) from adult bone marrow of each group. Cells were stained with anti-mouse Ter119-FITC, anti-mouse CD71-PE (BD-Bioscience, San Jose, CA, USA), and anti-Hb γ-PE (Santa Cruz Biotechnologies, Dallas, TX, USA) and anti-HBD-APC antibodies (Lifespan Biosciences, Newark, CA, USA) at a final concentration of 1:100 for 20 min at 4 °C in the dark. Specifically for the intracellular staining of HBG and HBD, cells were fixed and permeabilized prior to antibody incubation using the Cytofix/Cytoperm Kit (BD Biosciences, San Jose, CA, USA) according to the manufacturer’s protocol. After incubation, cells were washed in phosphate-buffered saline (PBS) containing 5% bovine serum albumin (BSA) and resuspended in FACS flow solution (BD Biosciences, San Jose, CA, USA). Data were acquired using a FACSCanto cytometer (BD Biosciences) and data analyzed with Flowjo version 10.8.1 (BD-Bioscience, San Jose, CA, USA). Each analysis was conducted on at least three mice.

### 2.5. Erythrocytes Morphology

Peripheral blood smears were obtained from mice and stained through RAL-555 kit according to the manufacturer’s protocol. Reticulocyte counts were performed on separate peripheral blood smears stained with Giemsa (azure–eosin–methylene blue) [59] according to standard laboratory procedures. At least 1000 RBCs per sample were examined across randomly selected microscopic fields, and frequency was expressed as a percentage of total erythrocytes. RBC subtypes and reticulocyte counts were assessed using a Leica DMIRE2-TCS-SL microscope (Leica, Wetzlar, Germany) and visualized using Leica Application Suite X software (Leica).

### 2.6. Liver and Spleen Iron Content and Weight

The liver and spleen were collected from mice in each group and weighed. Spleens were photographed for gross morphological analysis. Non-heme iron content was quantified using the established method of Torrance and Bothwell [60]. Briefly, tissue samples were homogenized and incubated in an acid solution at 65 °C for 48 h. The acid-digested solutions were then mixed with a chromogenic reagent, and iron concentration was determined by measuring the absorbance at 535 nm using a Nanodrop 2000C Spectrophotometer (Thermo Fisher Scientific, Boston, MA, USA).

### 2.7. Western Blot

Proteins were extracted from whole bone marrow (negative control: HEK293 cells) and peripheral blood (negative control: HeLa cells and C57BL/6 peripheral blood). For peripheral blood samples, proteins were precipitated with cold acetone and resuspended prior to quantification. Protein concentration was measured by Bradford assay. To confirm antibody specificity in bone marrow samples, a higher amount of HEK293 lysate (50 µg) was loaded compared to bone marrow samples (20 µg). Proteins were separated on a 4–12% SDS-PAGE gel and transferred to a PVDF membrane (26 V, overnight). The membrane was blocked with 5% milk in TBST for 1 h and incubated with primary antibodies: anti-Calnexin (Santa Cruz Biotechnologies, Dallas, TX, USA) (1:100, overnight), anti-β-actin (Santa Cruz Biotechnologies, Dallas, TX, USA. 1:1000, O/N), anti-HBD (HBD polyclonal antibody, Proteintech, Manchester, UK) (1:1000, 1 h), and anti-HBG1/2 (Novus Biotechnologies, Minneapolis, MN, USA) (1:1000, 1 h). After washing, incubation with HRP-conjugated secondary antibody (1:4000, 1 h) was performed. Detection used ECL Prime on photographic films. Densitometric analysis of peripheral blood bands was performed using ImageJ software.

### 2.8. Statistical Analysis

Data are presented as mean ± standard deviation. For comparisons between two biological groups in independent experiments, each including three animals per group, a two-tailed *t*-test was applied. Independent replicates were used to confirm the consistency of the results, while the single experiment provided the primary dataset. For experiments with a single set of animals, a one-way ANOVA followed by Tukey’s post hoc test was employed to evaluate differences between the three groups, whereas in experiments conducted in triplicate, ANOVA was applied to compare group means while accounting for variability across independent replicates. For experiments analyzed by ANOVA, Tukey post hoc tests were applied to compare group means, while for comparisons between two groups, significance was assessed directly using the *t*-test.

## 3. Results

### 3.1. Establishing of a Ccnd3-Deficient Humanized Mouse Model of β-Thal

**Figure 1:**
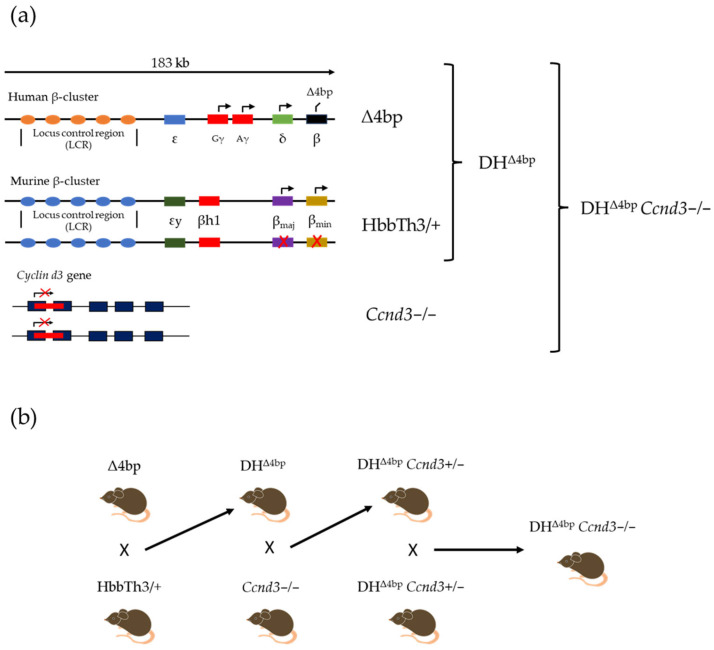
(**a**) Schematic representation of genetic editing in the three mouse lines: Δ4bp transgene containing the human β-cluster and the mutation in the β-globin gene determining its silencing; Murine β-cluster with the depletion of both β genes in heterozygosity to obtain Hbbth3/+ mice; KO of the *Ccnd3* gene. DH^Δ4bp^ mice are obtained by breeding of Δ4bp and Hbbth3/+ mice and DH^Δ4bp^ *Ccnd3−/−* by breeding DH^Δ4bp^ with DH^Δ4bp^ *Ccnd3+/−*. (**b**) Breeding strategy to obtain the three groups of mice analyzed in the project: DH^Δ4bp^ (*Ccnd3+/+*) as the control group, *Ccnd3+/−*, and *Ccnd3−/−*. DH: double heterozygotes; KO: knock out. Red crosses indicate gene silencing.

An experimental *Ccnd3*-deficient humanized mouse model of β-thal was established through sequential breeding of three mouse lines (Figure 1a). The first line, named Δ4bp, is a transgenic model that, in heterozygosity, carries six copies of a 183-kilobase (kb) genomic fragment containing the entire human β-globin gene cluster [61,62,63,64]. The β-globin gene cluster contains a common mutation in southern China and Thailand caused by a 4 bp deletion (-TTCT) in codons 41–42 of the *HBB* gene [MIM: 141900], resulting in the loss of human β-globin gene expression [65,66], while the expression of the other β-like genes remains unaffected (Figure 1a) [57]. The second line, named Hbbth3/+, is a knockout (KO) mouse model of β-thal in which both the β minor and major genes (b1 and b2) of the murine adult globin genes are deleted (Figure 1a) [58]. Mice heterozygous for this mutation exhibit a phenotype that, while resembling human NTDT (Hb levels in the range of 8 to 9 g/dL), also exhibits some characteristics typical of severe β-thal [58]. Finally, the third line, named *Ccnd3−/−*, exhibits a deletion of the *Ccnd3* gene (Figure 1a) [56].

To obtain the target genotypes and relative controls, we first bred Δ4bp and Hbbth3/+ mice, obtaining a double heterozygous line, named DH^Δ4bp^ (DH^Δ4bp^ *Ccnd3+/+*), which represents our control group and has been previously described by Iannou’s lab [57]. Subsequently, DH^Δ4bp^ mice were mated to *Ccnd3−/−* mice obtaining DH^Δ4bp^ *Ccnd3+/−* mice which were inter-crossed to obtain our target group, DH^Δ4bp^ *Ccnd3−/−* (Figure 1b).

### 3.2. Ccnd3 Deprivation Leads to Increased Hb Levels

**Figure 2:**
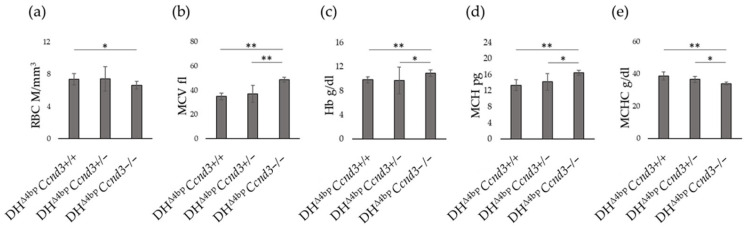
Comparison of (**a**) RBC, (**b**) MCV, (**c**) Hb, (**d**) MCH, (**e**) MCHC parameters between DH^Δ4bp^, DH^Δ4bp^ *Ccnd3*+/−, and DH^Δ4bp^ *Ccnd3*−/− mice. Data were obtained from 14 animals per group (DH^Δ4bp^: eight females, six males; DH^Δ4bp^ *Ccnd3+/−*: seven females, seven males; DH^Δ4bp^ *Ccnd3−/−*: six females, eight males; age range 4–8 months). The error bars represent the standard deviation. (*p*-value: * < 0.05; ** < 0.01). Hb: hemoglobin; MCH: mean corpuscular hemoglobin; MCHC: mean corpuscular hemoglobin concentration; MCV: mean corpuscular volume; RBC: red blood cells.

Haematological parameters confirmed in β-thal mice the impact of *Ccnd3* shortage on RBC numbers and volume that was previously observed in non-thalassaemic mice [46,56]. Mice were within a comparable age range (4–8 months) and sex-balanced across groups. Compared to DH^Δ4bp^ controls, the RBC count in DH^Δ4bp^ *Ccnd3−/−* decreased by 9% (*p*-value: <0.05) (Figure 2a), while the MCV increased by 28.43% (*p*-value: <0.01) (Figure 2b). Notably, total Hb increased by 10.15% (*p*-value: <0.01) in DH^Δ4bp^ *Ccnd3*−/− mice compared to DH^Δ4bp^, the entity of this increase being of approximately 1 g/dL of Hb (Figure 2c). The MCH parameter was 18.7% higher in DH^Δ4bp^ *Ccnd3*−/− mice than in DH^Δ4bp^ mice, while the mean corpuscular hemoglobin concentration (MCHC) parameter was 12.9% (*p*-value: <0.01) lower (Figure 2d,e).

Analysis of DH^Δ4bp^ *Ccnd3*+/− mice revealed substantial homogeneity with DH^Δ4bp^ controls; no significant differences were observed. Furthermore, comparison of DH^Δ4bp^ *Ccnd3*+/− and DH^Δ4bp^ *Ccnd3*−/− mice showed significant differences in MCV (plus 24.30% in DH^Δ4bp^ *Ccnd3*−/−; *p*-value: <0.01) (Figure 2b), Hb (plus 11.08% in DH^Δ4bp^ *Ccnd3*−/−; *p*-value: <0.01) (Figure 2c), MCH (plus 13.66% in DH^Δ4bp^ *Ccnd3*−/−: *p*-value: <0.05) (Figure 2d) and MCHC (minus 7.21% in DH^Δ4bp^ *Ccnd3*−/−: *p*-value: 0.022) (Figure 2e).

Overall, these data are milder but consistent with those previously reported for the mouse model lacking *Ccnd3* expression [46,56]. Notably, however, we observed a significant increase in Hb levels in DH^Δ4bp^ *Ccnd3−/−* mice compared to DH^Δ4bp^ mice, indicating an improvement of the anaemia.

### 3.3. γ- and δ-Globin Expression in DH^Δ4bp^ Ccnd3−/− Mouse Model

**Figure 3:**
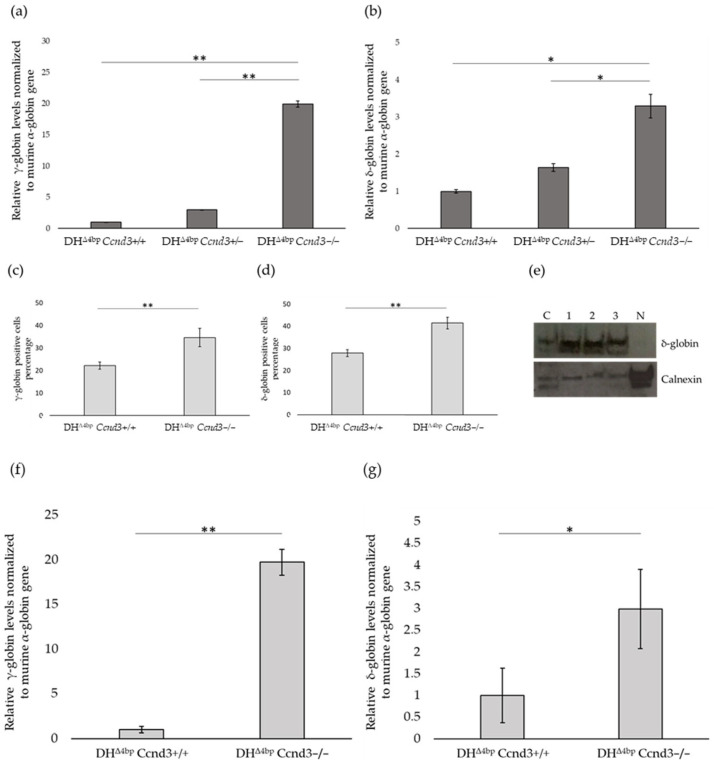
(**a**) Levels of expression γ-globin transcript obtained by qPCR analysis of bone marrow cells from the three groups of mice (DH^Δ4bp^, DH^Δ4bp^ *Ccnd3*+/−, and DH^Δ4bp^ *Ccnd3*−/−). (**b**) qPCR analysis of δ-globin transcript. (**c**) Bar plot representing the percentages of flow cytometry analysis of γ-expressing cells in the bone marrow of DH^Δ4bp^ and DH^Δ4bp^ *Ccnd3*−/−. (**d**) Percentages of δ-expressing cells. Each data point from (**c**,**d**) represents the mean of three independent experiments (with at least three mice per experiment; age: 4–8 months; DH^Δ4bp^: F:6, M:3; DH^Δ4bp^ *Ccnd3*−/−: F:5, M:4). Error bars indicate the standard deviation. (*p*-value: * < 0.05; ** < 0.01). (**e**) Western blot analysis of δ-globin levels: C: control (DH^Δ4bp^ *Ccnd3+/+*); 1, 2, 3: three different DH^Δ4bp^ *Ccnd3*−/− samples from different mice; N: negative control (HEK293 cells). Anti-Calnexin antibody was used as an endogenous control. The image is representative of three independent experiments. (**f**) Levels of γ-globin transcript obtained by qPCR analysis of peripheral blood cells from DH^Δ4bp^ and DH^Δ4bp^ *Ccnd3*−/− mice. (**g**) Levels of δ-globin transcript obtained by qPCR analysis of the same peripheral blood cells. For (**a**,**b**,**f**,**g**), mRNA expression data were normalized to mouse α-globin and are presented as the fold change relative to the control. Analyses were performed in three independent experiments using comparable groups of mice, within a similar age range (4–8 months) and with balanced sex distribution (DH^Δ4bp^: F:6, M:3; DH^Δ4bp^ *Ccnd3*+/−: F:4, M:5; DH^Δ4bp^ *Ccnd3*−/−: F:5, M:4). Error bars represent the normalized standard deviation. (*p*-value: * < 0.05; ** < 0.01).

To verify if the increase in total Hb observed in DH^Δ4bp^ *Ccnd3−/−* mice was determined by the rise of γ- and δ-globin expression, bone marrow from DH^Δ4bp^, DH^Δ4bp^ *Ccnd3+/−*, and DH^Δ4bp^ *Ccnd3−/−* mice was collected and globin expression analyzed through quantitative PCR (qPCR). Data were normalized relative to murine α-globin, which was previously verified to be unaffected by *Ccnd3* deletion (Figure S1a,b). Analysis of γ-globin showed an increase of about 20 fold in DH^Δ4bp^ *Ccnd3−/−* mice compared to controls (19.95 ± 0.31; *p*-value: <0.01) (Figure 3a). Similarly, δ-globin transcript analysis in DH^Δ4bp^ *Ccnd3*−/− mice showed more than a 3-fold increase compared to DH^Δ4bp^ (3.29 ± 0.67, *p*-value: <0.05) (Figure 3b). No significant differences in γ- and δ-globin levels were observed between DH^Δ4bp^ *Ccnd3+/−* and DH^Δ4bp^ mice (Figure 3a,b), indicating that *Ccnd3* heterozygosity in the DH^Δ4bp^ background does not produce detectable effects compared with DH^Δ4bp^ controls; this is consistent with previous findings [46] and therefore DH^Δ4bp^ *Ccnd3+/−* animals were not analyzed further.

Finally, no significant differences were observed in mutant human β-globin or murine β-globin expression between DH^Δ4bp^ and DH^Δ4bp^ *Ccnd3−/−* mice (Figure S2a,b).

Expression of γ- and δ-globins was further analyzed by flow cytometry. Bone marrow freshly isolated cells from DH^Δ4bp^ and DH^Δ4bp^ *Ccnd3*−/− adult mice were labelled for Ter119 to identify erythroid cells, permeabilized, and labelled for anti-γ and anti-δ antibody. Data showed a significant increase in γ- (DH^Δ4bp^: 22.2% ± 1.57; DH^Δ4bp^ *Ccnd3*−/−: 34.63% ± 4.05; *p*-value: 0.007) (Figure 3c) and δ-(DH^Δ4bp^ *Ccnd3*+/+: 27.86% ± 1.55; DH^Δ4bp^ *Ccnd3*−/−: 41.46% ± 2.63; *p*-value: 0.0015) (Figure 3d) positive cells in mice lacking *Ccnd3* expression compared to controls. To further confirm the increase in γ and δ globin, we performed a western blot assay on bone marrow cells with anti-γ and anti-δ antibodies. Comparison of three independent DH^Δ4bp^ *Ccnd3−/−* mice with one DH^Δ4bp^ mouse revealed a marked increase in δ-globin expression (plus 3.64 ± 0.73 in DH^Δ4bp^ *Ccnd3*−/−; *p*-value: 0.0009) (Figure 3e and Figure S3a). Surprisingly, we were unable to detect γ-globin expression by western blot assay. This unexpected finding raises the possibility that, although γ-globin expression is increased in *Ccnd3*-deficient mice, overall expression levels in Δ4bp and DH^Δ4bp^ mice remain too low for this increase to translate into a meaningful measurable effect by western blotting. Therefore, we quantified relative globin expression by qPCR and calculated the relative proportion of each β-globin to total beta-like globin in Δ4bp, DH^Δ4bp^, and DH^Δ4bp^ *Ccnd3−/−* mice. γ-globin increased progressively from 0.0016% in Δ4bp to 0.0033% in DH^Δ4bp^ and to 0.053% in DH^Δ4bp^ *Ccnd3−/−* mice, expressed as a percentage of the total β-like globin pool (Figure S3b). δ-Globin increased from 1% to 1.7% and 4.5% across the same groups (Figure S3c).

To assess whether the increase in globin transcripts observed in bone marrow was also present in circulating RBC, we performed qPCR on blood samples from DH^Δ4bp^ and DH^Δ4bp^ *Ccnd3−/−* mice. γ-globin transcripts were elevated by approximately 20-fold in DH^Δ4bp^ *Ccnd3−/−* mice compared to DH^Δ4bp^ controls (19.72 ± 1.46, *p*-value: 0.0028) (Figure 3f), while δ-globin levels increased by about 3-fold relative to controls (2.98 ± 0.91, *p*-value: 0.029) (Figure 3g). Western blot analysis and relative densitometry confirmed increased δ-globin levels (Figure S4a,b), whereas γ-globin was undetectable, consistent with the bone marrow finding of a very low basal expression.

Overall, these data demonstrate a robust relative increase in γ- and δ-globin expression (fold change versus control) in DH^Δ4bp^ *Ccnd3*–/– mice, likely contributing to the observed elevation in total Hb levels. Nonetheless, the low basal expression of γ-globin—and, to a lesser extent, δ-globin—in Δ4bp and DH^Δ4bp^ mice resulted in a modest contribution to total globin synthesis, thereby substantially limiting their overall impact on total Hb elevation as a result of *Ccnd3* deprivation in this model.

### 3.4. Erythropoiesis and RBC Morphology in DH^Δ4bp^ Ccnd3−/− Mice

**Figure 4:**
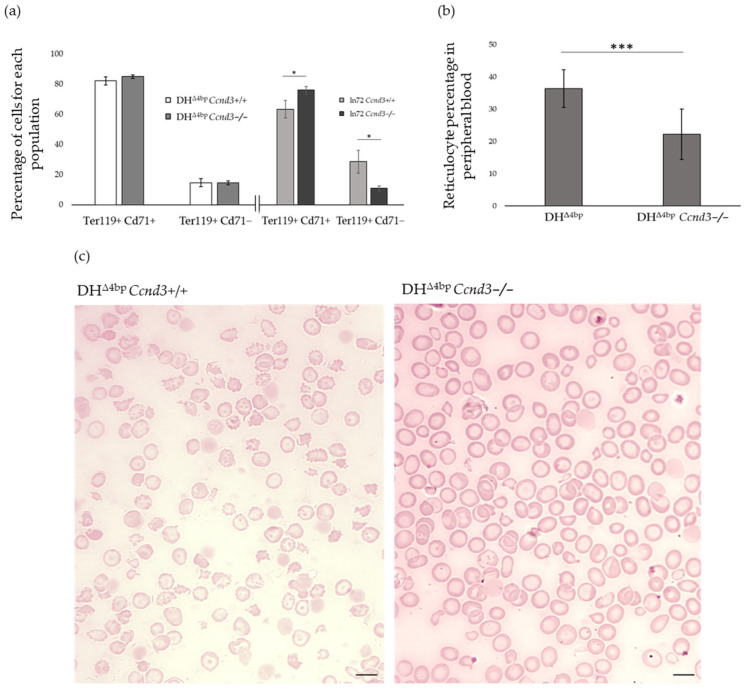
(**a**) Erythropoiesis in DH^Δ4bp^ *Ccnd3*+/+ and DH^Δ4bp^ *Ccnd3*−/− mice as obtained by analyzing Ter119 and Cd71 markers by flow cytometry (left). Each datum has been obtained from at least five mice (age: 4–8 months) with balanced sex distribution (DH^Δ4bp^: F: 3, M: 2; DH^Δ4bp^ *Ccnd3*−/−: F: 2, M:3). Error bars indicate the standard deviation (*p*-value: * < 0.05). On the right, data from the previously published non-thalassemic mouse model are shown for comparison [46] (**b**) Reticulocyte counts expressed as percentage of total erythrocytes in DH^Δ4bp^ and DH^Δ4bp^ *Ccnd3−/−* mice. Data were obtained from five animals per group (DH^Δ4bp^: three females, two males; DH^Δ4bp^ *Ccnd3−/−*: two females, three males; age range 4–8 months) (*p*-value: *** < 0.001). The error bars represent the standard deviation. (**c**) Blood smears images from DH^Δ4bp^ *Ccnd3*+/+ and DH^Δ4bp^ *Ccnd3*−/− mice. Images are representative of observation from five animals per group (DH^Δ4bp^: three females, two males; DH^Δ4bp^ *Ccnd3−/−*: two females, three males; age range 4–8 months), magnification = ×, scale bar = 8 μm.

Freshly isolated bone marrow cells from DH^Δ4bp^ and DH^Δ4bp^ *Ccnd3*−/− mice were labelled with anti-Ter119 and anti-CD71 antibodies [67] and analyzed by flow cytometry to verify the effects of *Ccnd3* expression on erythroblast differentiation. This analysis revealed no significant differences in the percentages of Ter119+ CD71+ erythroblasts (comprising proerythroblasts, basophilic, and polychromatic normoblasts) or the more mature Ter119+ CD71- population (mainly orthochromatic normoblasts and reticulocytes) (Figure 4a).

The apparent absence of substantial differences in erythroblast differentiation could be interpreted as evidence that *Ccnd3* loss does not ameliorate IE. However, when analyzed by flow cytometry, the effects of *Ccnd3* absence on the cell cycle alter erythroid differentiation kinetics in a manner which overlaps with that produced by IE, potentially masking any improvement. This is in line with our previously reported data in non-thalassemic mice (Figure 4a) [46]. The attempts to obtain a Hbbth3/+ *Ccnd3−/−* model, as a control for this hypothesis, resulted in in utero lethality (0 out of 187 genotyped pups, versus an expected 25%). This lethality suggests that the combined effects of β-thal-associated IE and the *Ccnd3−/−* mitotic block are non-viable in the absence of γ- and δ-globin expression. We then compared erythropoiesis in Hbbth3/+, *Ccnd3−/−* and DH^Δ4bp^ *Ccnd3−/−* mice by flow cytometry (Figure S5). The differentiation pattern of the DH^Δ4bp^ *Ccnd3−/−* model was phenotypically inseparable from that of *Ccnd3−/−* mice, indicating that the pattern mirrors a non-pathological, cell cycle-driven phenotype. Moreover, despite a slight but significant difference, the overall differentiation pattern of both *Ccnd3−/−* and DH^Δ4bp^ *Ccnd3−/−* mice remained strikingly similar to Hbbth3/+ mice. This outcome confirms the striking similarity between the differentiation pattern caused by *Ccnd3* loss and the β-thal phenotype (*Ccnd3−/−* versus Hbbth3/+), suggesting that the DH^Δ4bp^ *Ccnd3−/−* phenotype emerges from the integration of these two overlapping conditions.

Collectively, these observations suggest that the pattern of erythroblast differentiation detected by flow cytometry masks an actual improvement in IE, as suggested by increased δ- and γ-globin expression as well as other indices (see below).

To test our hypothesis, we examined blood smears from DH^Δ4bp^ and DH^Δ4bp^ *Ccnd3−/−* mice by light microscopy to quantify reticulocytes levels in peripheral blood. DH^Δ4bp^ mice exhibited 36.38 ± 7.84% reticulocytes (Figure 4b), consistent with previous reports for this model [57]. In contrast, DH^Δ4bp^ *Ccnd3−/−* mice showed a significantly lower percentage (22.25 ± 7.78%), representing a 42.49% reduction relative to control mice (*p* = 0.00078).

Observation of peripheral blood smears from DH^Δ4bp^ mice confirmed the typical signs of β-thal (Figure 4c). The RBCs showed anisocytosis, poikilocytosis, and hypochromia, as well as several target cells, schistocytes, and Burr cells. DH^Δ4bp^ *Ccnd3*−/− erythrocytes, however, showed an evident improvement of their morphology (Figure 4c). Hypochromia and anisocytosis were still present but at a lower frequency compared to DH^Δ4bp^ controls. RBCs showed an evident increase in their volume in agreement with the *Ccnd3*−/− phenotype. Moreover, numbers of target cells, or other alterations of erythrocyte morphology, were strongly reduced in comparison to DH^Δ4bp^ mice (Figure S6a–c).

In general, these data highlight appreciable improvement in reticulocyte count and RBC morphology in the peripheral blood of mice lacking *Ccnd3* expression (DH^Δ4bp^ *Ccnd3*−/−), consistent with a likely increase in red blood cell lifespan.

### 3.5. Ccnd3 Deficiency Decreases Iron Content and Alleviates Hepatosplenomegaly

**Figure 5:**
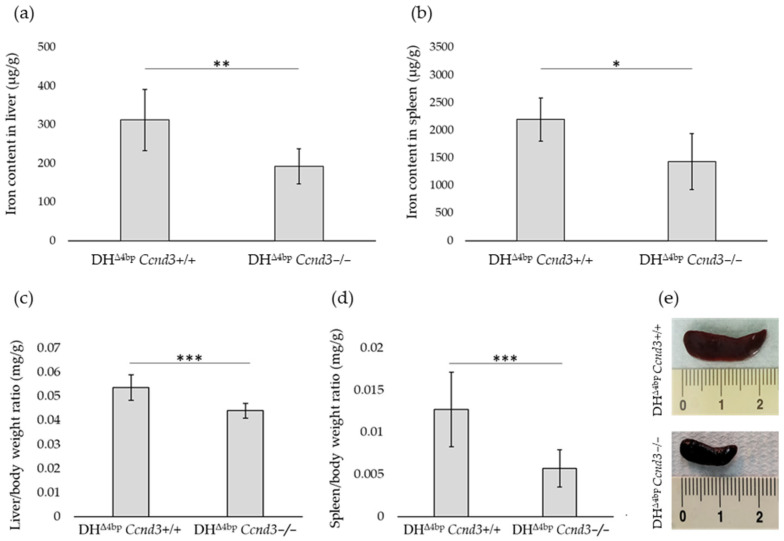
(**a**) Comparison of iron content in livers of DH^Δ4bp^ and DH^Δ4bp^ *Ccnd3−/−* mice, data are shown as the ratio of iron (μg) to organ tissues (g). (**b**) Same comparison of iron content in the spleen of DH^Δ4bp^ and DH^Δ4bp^ *Ccnd3−/−* mice. (**c**) Ratio of liver (mg) to mice body weight (g) in DH^Δ4bp^ and DH^Δ4bp^ *Ccnd3−/−* mice. (**d**) Ratio of spleen (mg) to mice body weight (g) in DH^Δ4bp^ Ccnd3+/+ and DH^Δ4bp^ *Ccnd3−/−* mice. (**e**) Comparative spleen volume of DH^Δ4bp^ *Ccnd3+/+* and DH^Δ4bp^ *Ccnd3−/−* mice. Analyses were performed in three independent experiments using comparable groups of mice. Each datum is representative of three independent experiments (at least three mice in each experiment). Animals were within a similar age range (4–8 months) and had a balanced sex distribution (DH^Δ4bp^: F = 6, M = 3; DH^Δ4bp^ *Ccnd3−/−^−^*: F = 5, M = 4). Error bars represent the standard deviation (*p*-value: * < 0.05; ** < 0.01; *** < 0.001). DH: double heterozygotes.

We evaluated the iron content of organs involved in RBC production and iron homeostasis by non-haeme iron quantification [68] in DH^Δ4bp^ and DH^Δ4bp^ *Ccnd3−/−* mice. A significant decrease in iron content was observed in DH^Δ4bp^ *Ccnd3−/−* mice in liver and spleen compared to the DH^Δ4bp^ mice organs (minus 38.46%; *p*-value: 0.0082 and minus 34.60; *p*-value: 0.017, respectively) (Figure 5a,b).

Analysis of liver and spleen weight from DH^Δ4bp^ and DH^Δ4bp^ *Ccnd3−/−* mice was also estimated. Both organs showed a significant reduction in the g organ/g body ratio. Liver weight decreased from 0.053 ± 0.0016 to 0.044 ± 0.001 (*p*-value: 0.00033) (Figure 5c) while spleen decreased from 0.0127 ± 0.0017 to 0.0057 ± 0.00073 (*p*-value: 0.005) in β-thal mice lacking *Ccnd3* expression (Figure 5d).

In agreement with these data, the collection of spleens from DH^Δ4bp^ and DH^Δ4bp^ *Ccnd3−/−* mice revealed a drastic reduction in volume in the DH^Δ4bp^ *Ccnd3−/−* group, where spleen size was nearly halved compared to controls (Figure 5e).

In β-thal, iron excess in organs and enlargement of the organs involved in erythrocyte homeostasis are consequent and proportional to the IE degree [13,14]. Therefore, the amelioration of these parameters supports a consistent improvement of IE in DH^Δ4bp^ *Ccnd3−/−*mice.

### 3.6. Loss of Ccnd3 Alters Key Mediators of Erythroblast Dysregulation and Improves a/b Ratio

**Figure 6:**
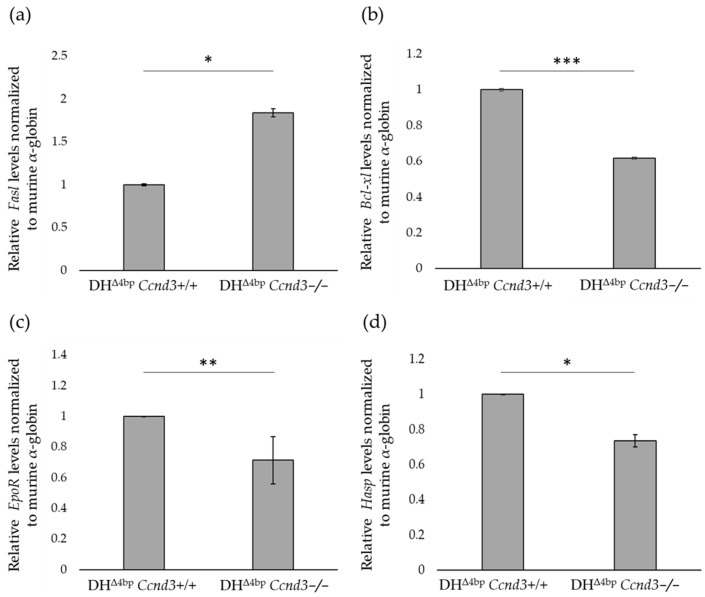
(**a**) *Fasl*, (**b**) *Bcl-xl*, (**c**) *Epor*, and (**d**) *Ahsp* gene levels from bone marrow of DH^Δ4bp^ and DH^Δ4bp^ *Ccnd3−/−* mice, data are normalized to mouse α-globin and indicated as fold change in mRNA expression relative to DH^Δ4bp^ control values. Analyses were performed in three independent experiments using comparable groups of mice, within a similar age range (4–8 months) and with balanced sex distribution (DH^Δ4bp^: F:6, M:3; DH^Δ4bp^ *Ccnd3+/−*: F:4, M:5; DH^Δ4bp^ *Ccnd3−/−*: F:5, M:4). The error bars represent the normalized standard deviation (*p*-value: * < 0.05; ** < 0.01; *** < 0.001). DH: double heterozygotes.

In β-thal, IE originates from a profound dysregulation of erythroid maturation. Current evidence indicates that IE is primarily driven by uncontrolled, ineffective proliferation and differentiation of erythroblasts, rather than apoptosis alone. This pathological state is largely determined by heightened erythropoietin (EPO) signalling. Consequently, this leads to an apparently paradoxical effect where physiological apoptosis is broadly suppressed, creating a permissive environment for the accumulation of defective erythroid precursors. This is exemplified by a paradoxical regulation of key apoptotic mediators: the suppression of the pro-apoptotic *Fas/Fas-ligand* (*Fas/Fasl*, [MIM: 134637, 134638]), which would normally increase to constrain population growth through apoptosis [67], alongside the upregulation of the anti-apoptotic *Bcl2 like 1* (*Bcl-XL*, [MIM*:* 600039]), which would normally decrease to allow for the removal of defective cells [69,70]. In the thalassaemic microenvironment, however, these combined actions foster pathological erythroblast expansion rather than effective RBC production. We therefore quantified the expression of these two key regulators in the bone marrow of DH^Δ4bp^ and DH^Δ4bp^ *Ccnd3−/−* mice to determine whether loss of *Ccnd3* impacts this pathogenic circuitry. Consistent with this dysregulated environment, *Fas-l* expression was significantly increased by 1.839-fold (*p*-value: 0.012) in DH^Δ4bp^ *Ccnd3−/−* mice compared to controls (Figure 6a). In contrast, *Bcl-XL* expression was significantly decreased, measuring 0.617-fold lower (*p*-value: 0.00022) in DH^Δ4bp^ *Ccnd3−/−* mice compared to normalized DH^Δ4bp^ controls (Figure 6b). To further investigate erythroblast differentiation potential, we analyzed *Erythropoietin receptor* (*Epor* [MIM: 133171]) expression levels by qPCR in bone marrow cells from DH^Δ4bp^ and DH^Δ4bp^ *Ccnd3−/−* mice. *Epor* is primarily expressed by immature erythroid precursors [71,72,73], and its level of expression is regulated by Epo [74,75,76,77]. We observed a decrease in *Epor* expression in DH^Δ4bp^ *Ccnd3−/−* mice of 0.713-fold compared to DH^Δ4bp^ controls (*p*-value: 0.0024, Figure 6c). This decrease is consistent with an amelioration of IE.

Moreover, to determine whether the absence of *Ccnd3* directly affects *Fasl*, *Bcl-xl*, or *Epor* expression, we performed qPCR on bone marrow cells of a non-thalassemic mouse model (ln72 and ln72 *Ccnd3−/−*). No significant differences were detected between the two groups for any of the genes analyzed (Figure S7a–c).

Taken together, these results suggest that in the DH^Δ4bp^ mouse model, the loss of *Ccnd3* contributes to a restoration of the physiological erythroid differentiation program.

Considering the overall amelioration of thalassaemic phenotype and IE in mice lacking *Ccnd3* expression, we investigated whether this improvement could be determined, at least in part, by a restoration in the α/β ratio. To test this, we determined by qPCR the levels of expression of *Alpha hemoglobin stabilizing protein* (*Ahsp*, [MIM: 605821]) gene in DH^Δ4bp^ and DH^Δ4bp^ *Ccnd3−/−* mice. Ahsp is a chaperone protein implicated in the stabilization of free α-globin chains, and its expression is proportional to the levels of unbound α-globin [78]. We found that *Ahsp* transcript levels were significantly lower in DH^Δ4bp^ *Ccnd3−/−* mice (0.73-fold, *p*-value: 0.015) compared to controls (Figure 6d).

This outcome suggests a reduced accumulation of free α-globin chains in *Ccnd3*-deficient erythroblasts.

## 4. Discussion

This study advances our investigation into the *CCND3* gene as a potential therapeutic target for β-thal and related β-hemoglobinopathies. Association of the *CCND3* gene with HbF and HbA2 levels [44] has been recently validated in a transgenic mouse model carrying the entire human β-globin cluster [45,46]. Furthermore, we previously provided evidence indicating that augmentation of γ- and δ-globin levels can be achieved through the administration of molecules, in particular palbociclib, a potent inhibitor of the kinases CDK4 and CDK6. The inhibition of CDK4 and CDK6 by Palbociclib results in a phenotype that is analogous to that observed in *Ccnd3*-null mice [47]. These data prompted us to test the effects of *Ccnd3* deprivation in a mouse model of human β-thal [57] to evaluate the potential therapeutic effect of this approach on a preclinical in vivo setting.

Our data confirmed that silencing the *Ccnd3* gene considerably increased the expression of γ- and δ-globin, with mRNA levels rising approximately 20-fold and 3-fold, respectively. Nevertheless, the absolute extent of the β-like globin increases results in a modest increment of the total Hb levels in this model (up to 1 g/dL). However, Hb levels directly correlate to erythrocyte count, which is significantly reduced in *Ccnd3* KO mouse models. Similarly, the reduction in the number of erythrocytes in humans has been associated with variants mapped in the *CCND3* locus [51,52,53,54,55]. Thus, a similar impact on RBC counts by modulating the gene is not unexpected.

A limitation of the mouse model employed in this study lies in its basal expression of γ- and δ-globin in adult animals. Nevertheless, to the best of our knowledge, the Δ4bp model remains the only one described to date [79,80,81,82] that fulfils the intrinsic requirements of our investigation—namely, the presence of a β^0^-thalassaemic mutation within the context of an intact human β-globin cluster. This configuration enables the simultaneous assessment of γ- and δ-globin expression and their impact on the β-thal phenotype. Nonetheless, although the baseline expression levels of γ- and δ-globin in the Δ4bp model are low and despite the known reduction in RBC associated with the *Ccnd3* KO phenotype, we observed a significant increase in total Hb upon *Ccnd3* depletion in a murine β-thal model.

These finding gains relevance when considered in a human context. Basal γ- and δ-globin expression in healthy humans is markedly higher than in Δ4bp mice and is further elevated in β-thal carriers, who typically exhibit HbF and HbA_2_ levels of approximately 0.1 and 0.7 g/dL (0.9% and 5.8%), respectively [44]. By extrapolating the fold changes observed in the Δ4bp model, one can estimate potential increases of ~2 g/dL in HbF- and ~2.3 g/dL in HbA2, leading to a total Hb gain exceeding 4 g/dL. Even when accounting for the approximate 9% reduction in RBC observed in *Ccnd3*-deficient mice, the net Hb increase could approach 3.5–3.6 g/dL. Nonetheless, these findings highlight the potential of pharmacological CCND3 modulation as a clinically relevant strategy to ameliorate anaemia in β-thal, possibly in combination with other fetal Hb inducers. Given species-specific differences in gene regulation, studies in a human context are needed to evaluate the feasibility of this approach. Moreover, although the *Ccnd3* KO phenotype is not pathological, its associated reduction in RBC numbers warrants further investigation in patient-derived cells to assess the therapeutic implications.

A comparison of erythropoiesis in DH^Δ4bp^ and DH^Δ4bp^ *Ccnd3−/−* mice by flow cytometry analysis revealed no apparent differences in the final stages of erythroblast differentiation. Thus, *Ccnd3* deprivation, seemingly, has no beneficial effects on the IE in these mice. However, the combination of Hbbth3/+ and *Ccnd3* KO was lethal, implying that *Ccnd3* loss exacerbates pre-existing perturbation of erythropoiesis when γ- and δ-globin genes are absent. Thus, the erythroid phenotype observed in DH^Δ4bp^ *Ccnd3^−^/^−^* mice likely results from the combined impact of *Ccnd3* loss on both erythroblast expansion and globin gene expression.

Despite these complexities, several parameters point toward a more physiologic erythroid maturation in DH^Δ4bp^ *Ccnd3−/−* mice: (1) The absence of *Ccnd3* expression in DH^Δ4bp^ mice is associated with significantly lower iron levels in the liver and spleen. There is a direct correlation between iron content and IE, with improvements in erythropoiesis determining decreases in iron levels [13,14]; (2) The decrease in liver weight and, more markedly, in spleen weight and volume, suggest recovery from hepatosplenomegaly, which is landmark of IE; (3) The enhancement in the levels of expression of markers known to be involved in apoptosis and in the abnormal expansion of erythroblasts-*Fas-l* and *Bcl-xl*-which, in Hbbth3/+ mice, are characteristic of stress erythropoiesis; (4) Furthermore, observations of peripheral blood smears show a decrease in reticulocytes count and general improvement of erythrocytes morphology suggesting an amelioration of erythrocyte homeostasis.

The observed amelioration in *Ccnd3*-deficient mice supports an improvement of erythropoiesis, likely reflecting a reduced Epo-dependent stimulus. While the absence of *Ccnd3* does not affect the intrinsic ability of erythroblasts to respond to Epo [56], the decrease in EpoR levels provides an indicator of the overall erythroid responsiveness. Epo regulates expression of its own receptor [74,75,76,77] and *Epor* is expressed by early erythroblasts [71,72,73], which are typically expanded in IE. Therefore, the observed decrease in Epor could result from reduced circulating Epo levels associated with increased hemoglobin levels, but also from a relative reduction in early erythroid precursors, consistent with a shift toward more physiologic erythroid maturation.

In line with this interpretation, the reduction in circulating reticulocytes further supports a decrease in the expansion of the erythroid compartment and, therefore, a decreased release of immature erythroid cells into the peripheral blood.

**Figure 7:**
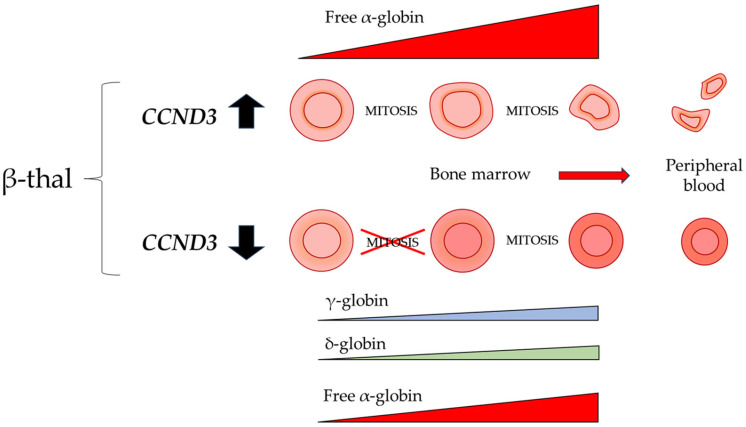
Schematic model of erythropoiesis and the effect of CCND3 deficiency. The diagram shows erythroid maturation from bone marrow (**left**) to peripheral blood (**right**). In the DH^Δ4bp^ model (**top**), excessive free α-globin causes IE, leading to damaged circulating cells. In the *Ccnd3* KO model (**bottom**), skipping one mitotic division combined with a compensatory increase in γ- and δ-globin ameliorates the α/β-like globin balance, limits free α-globin accumulation, and promotes more physiological erythroid maturation.

Collectively, the observed phenotypic amelioration appears disproportionate relative to the modest increase in total Hb. This could suggest that the benefits of *Ccnd3* deficiency in β-thal in our mouse model may extend beyond a simple boost in Hb production. An important point to consider, however, is the intrinsic erythroid effects of *Ccnd3* silencing, specifically given the role of *Ccnd3* in regulating the cell cycle. Perturbation of erythroid proliferation dynamics can influence erythroblast maturation kinetics and the timing of globin gene expression [83,84]. Likewise, *Ccnd3* deficiency results in a non-pathological alteration of erythropoiesis that results in higher Hb content per erythrocyte, as reflected by the increased MCH values. This is consistent with the observed pan-cellular increase in HbA2, coupled with an upregulation of γ-globin expression, and the partial restoration of the α/β-globin chain balance, the primary trigger for IE in β-thal patients (Figure 7). Therefore, a possible explanation is that this unique erythroid profile may be intrinsically more resilient to the burden of IE, thereby breaking the cycle of pathology and leading to broader systemic improvement.

Here, we aimed to further explore the contribution of *CCND3* by providing in vivo evidence of its impact on globin production and IE in a murine model of β-thal. By linking genetic association data to disease-relevant functional outcomes, this study contributes to a deeper understanding of the biological role of *CCND3* in erythropoiesis and to validating *CCND3* as a potential therapeutic target for the disease.

An aspect to consider in evaluating *CCND3* as a therapeutic target for β-thal is the adverse effects associated with prolonged use of CDK4/6 inhibitors. Cases of thrombosis were reported [85,86,87,88,89]. Nevertheless, recent evidence indicates that palbociclib induces an increase in γ-globin production in both primary human erythroid cells and the Townes SCD mouse model, at doses that are well tolerated in this model [90]. Moreover, the availability of multiple CDK4/6 inhibitors, including more recent compounds with potentially different safety profiles, together with the possible use of antithrombotic therapies in combination, may allow mitigation of thrombotic risk.

β-hemoglobinopathies pose substantial global health challenges. Although significant advances have been achieved with gene addition therapy and CRISPR/Cas9-based therapeutics, their accessibility remains limited in the countries where most affected individuals reside. Therefore, the development of innovative therapeutic approaches based on compounds that are both effective and affordable is of critical importance. In this context, drug repurposing and small-molecule screening using suitable assays are actively explored by multiple research groups and represent a key strategy for the discovery of new therapeutic options.

## 5. Conclusions

β-thal is a burden on healthcare systems worldwide. The outlook is expected to worsen, with regions where the condition was rare now experiencing higher incidence rates. While effective therapies exist, there is an increasing need to identify pharmacological targets for the development of affordable drugs that can be distributed worldwide.

We recently highlighted that the increase in g- and d-globin can be achieved by molecules inhibiting *Ccnd3* activation of CDK4 and 6 ex vivo, with an output similar to that obtained by completely silencing the gene [47].

Here, we have evaluated the impact of *Ccnd3* deprivation on a murine model of β-thal. Results show an improved anaemia, erythroblast homeostasis, and differentiation. Future preclinical studies evaluating the effects of *Ccnd3* pharmacological modulation in human erythroid culture from patients and in a humanized mouse model of β-thal are necessary to validate *CCND3*’s potential as a therapeutic target for the disease.

## Data Availability

The original contributions presented in this study are included in the article/Supplementary Materials. Further inquiries can be directed to the corresponding author.

## Supplementary Materials

The following supporting information can be downloaded at: https://www.mdpi.com/article/10.3390/cells15060495/s1. Table S1. List of oligos used in this work for genotyping and qPCR assay. Figure S1. α-globin transcript expression levels in DH^Δ4bp^ and DH^Δ4bp^ *Ccnd3−/−*. Analysis was performed on freshly isolated bone marrow cells from adult mice (4–8 months old) of both sexes. Data were normalized to murine β-actin mRNA levels (*p*-value: 0.867) and β2m (*p*-value: 0.986) mRNA levels. The error bars represent the normalized standard deviation. Analyses were performed in three independent experiments using comparable groups of mice, within a similar age range (4–8 months) and with balanced sex distribution (DH^Δ4bp^: F:6, M:3; DH^Δ4bp^ *Ccnd3−/−*: F:5, M:4). Figure S2. Relative expression levels of mutant (a) human and (b) murine β-globin transcript in bone marrow cells from DH^Δ4bp^ and DH^Δ4bp^ *Ccnd3−/−* adult mice, obtained by qPCR assay. mRNA expression data were normalized to mouse α-globin (*p* = 0.639 and 0.469). Analyses were performed in three independent experiments using comparable groups of mice, within a similar age range (4–8 months) and with balanced sex distribution (DH^Δ4bp^: F:6, M:3; DH^Δ4bp^ *Ccnd3−/−*: F:5, M:4). The error bars represent the normalized standard deviation. Figure S3. (a) Densitometric analysis of δ-globin bands from the Western blot shown in Figure 3e. Band intensities in DHΔ4bp *Ccnd3*−/− mice are expressed relative to the DHΔ4bp control and reported as relative densitometric units. Data represent the mean of three independent experiments; error bars indicate the normalized standard deviation (*p* = 0.0009). Analysis was performed using ImageJ software. (b) Levels of expression of (a) γ-globin and (c) δ-globin transcripts in bone marrow cells from Δ4bp, DHΔ^4bp^, and DH^Δ4bp^ *Ccnd3−/−* adult mice obtained by qPCR assay. mRNA expression data were normalized to mouse α-globin. Expression levels of γ- and δ-globin are presented as a percentage relative to total beta-like globins (human γ + human δ + murine β). The error bars represent the standard deviation. (*p*-value: * < 0.05). Analyses were performed in three independent experiments using comparable groups of mice, within a similar age range (4–8 months) and with balanced sex distribution (Δ4bp: F:5, M:4; DH^Δ4bp^: F:6, M:3; DH^Δ4bp^ *Ccnd3−/−*: F:5, M:4). Figure S4. (a) Representative western blot analysis of δ-globin chain expression in peripheral blood from adult DH^Δ4bp^ and DH^Δ4bp^ *Ccnd3−/−* mice. Lane 1–2: DH^Δ4bp^ mice; lane 3: DH^Δ4bp^ *Ccnd3−/−* mouse; lane 4: C57BL/6 wild-type peripheral blood (negative control); lane 5: HeLa cells (negative control). β-actin was used as an endogenous control. The blot shown is representative of three independent experiments. Animals were 4–8 months old and of comparable sex. (b) Densitometric analysis performed using ImageJ software. The graph shows δ-globin band intensities of DH^Δ4bp^ *Ccnd3−/−* mice relative to the mean of the two DH^Δ4bp^ controls, expressed as relative densitometric units. Error bars represent the normalized standard deviation between biological replicates (*p*-value: 0.00045). Figure S5. Flow cytometry analysis of erythropoiesis in Hbbth3/+, *Ccnd3−/−*, and DH^Δ4bp^ *Ccnd3−/−* bone marrow freshly isolated cells. Analysis was conducted considering Ter119 and Cd71 marker expression levels. At least three mice from each group within a similar age range (4–8 months) and with balanced sex distribution (Hbbth3/+: F:2, M:1; *Ccnd3−/−*: F:1, M:2; DH^Δ4bp^ *Ccnd3−/−*: F:2, M:1) were analyzed. The error bars represent the standard deviation. (*p*-value: * < 0.05; ** < 0.01). Figure S6. Frequency of erythrocytes with thalassemia-associated morphological features in DH^Δ4bp^ *Ccnd3*−/− mice, normalized to DH^Δ4bp^ controls. (a) Target cells (*p* = 0.043), (b) Schistocytes (*p* = 0.013), (c) Burr cells (*p* = 0.00076). Data were obtained from five animals per group (DHΔ4bp: three females, two males; DH^Δ4bp^ *Ccnd3*−/−: two females, three males; age range 4–8 months). The error bars represent the normalized standard deviation. Figure S7. Relative expression levels of (a) *Fasl* (*p* value: 0.74), (b) *Bcl-xl* (*p* value: 0.38), and (c) *Epor* (*p* value: 0.24) transcripts in bone marrow cells from ln72 and ln72 *Ccnd3*−/− adult mice, obtained by qPCR assay. mRNA expression data were normalized to mouse α-globin. Analyses were performed on five samples per group using comparable groups of mice with similar age range and sex distribution. The error bars represent the normalized standard deviation (*p* value).
