# Supplementary material for: CCND3 Suppression Ameliorates β-Thalassaemia in a Murine Disease Model: A Potential Therapeutic Strategy
Source: Cells. 2026 Mar 10;15(6):495. doi: 10.3390/cells15060495 (PMC13025363; doi:10.3390/cells15060495)
Supplement: Supplementary file 1 [file cells-15-00495-s001.zip › cells-4078569-supplementary.pdf]

Table S1. List of oligos used in this work for genotyping and qPCR assay

GENE	SPECIES	APPLICATION	PRIMER SEQUENCE (5’→3’)
Ccnd3	Mouse	Genotyping	TCCATCCTGCGATGGCTCAC<br>TGCTGTCCATCTGCACGAGA<br>GAACGTTGTGACGTAGGAGC
**HPRT**	Mouse	Genotyping	AGATGCTCATGGCCTCATTG<br>ACAGGTAACCAGGTCATTGC
**LUG**	Mouse	Genotyping	ACAAGACAGGTTTAAGGAGACCA<br>GTCTGTTTCCCATTCTAAACTGTA
**β-globin**	Human	RT-qPCR	TTGGACCCAGAGGTTCTTTGA<br>TCACTAAAGGCACCGAGCACT
**γ-globin**	Human	RT-qPCR	CTGAGTGAACTGCACTGTGACAAG<br>TCTTTGCCGAAATGGATTGC
**δ-globin**	Human	RT-qPCR	AGGTGCTAGGTGCCTTTAGTGA<br>GGGTGAATTCCTTGCCAAAGTTGC
**α-globin**	Mouse	RT-qPCR	CACCACCCTGCCGATTTC<br>CTCACAGAGGCAAGGAATTTGTC
**β-globin**	Mouse	RT-qPCR	TTTAACGATGGCCTGAATCACTT<br>CAGCACAATCACGATCATATTGC
**FASL**	Mouse	RT-qPCR	CTGGTGGCTCTGGTTGGAATG<br>GATGATACTTTAAGGCTTTGGTTG
Bcl2l1-XL	Mouse	RT-qPCR	CTTGCTGTCGCCGGAGATAG<br>GGATCCAAAGCCAAGATAAGGTTC
**AHSP**	Mouse	RT-qPCR	CTCAGCACCATTAGACTTGAAGAT<br>TGATCCAGCAGAACATTAAACTC
**Epor**	Mouse	RT-qPCR	GCTCCGGGATGGACTTCA<br>GAGCCTGGTGCAGGCTACAT
**β-actin**	Mouse	RT-qPCR	ACGGCCAGGTCATCACTATTG<br>CAAGAAGGAAGGCTGGAAAAG
**β2m**	Mouse	RT-qPCR	ATTCACCCCCACTGAGACTGA<br>CTCGATCCCAGTAGACGGTC

Table S1. List of oligos used in this work for genotyping and qPCR assay

Figure S1

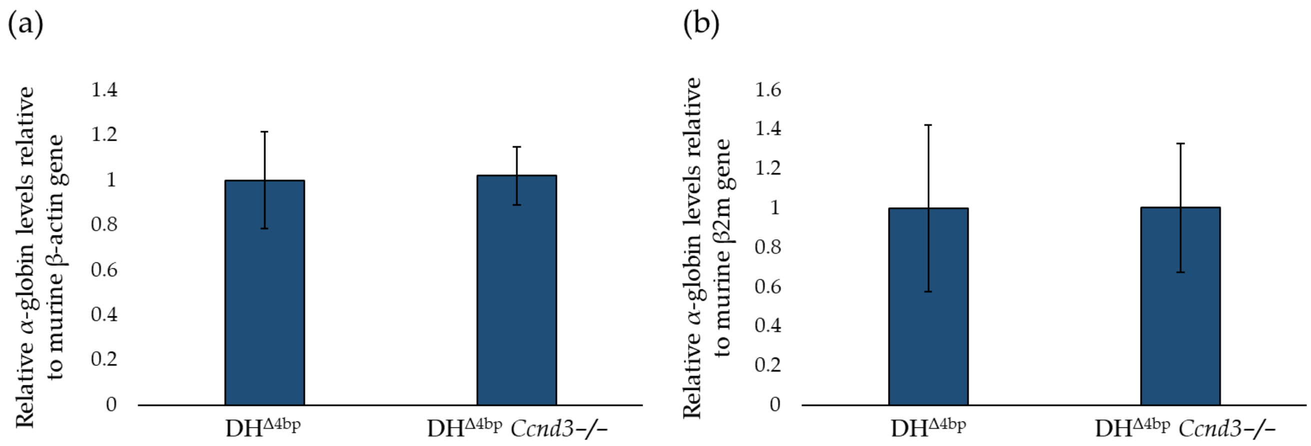

Figure S1. α-globin transcript expression levels in $DH^{\Delta 4bp}$ and $DH^{\Delta 4bp}$ *Ccnd3*-/-. Analysis was performed on freshly isolated bone marrow cells from adult mice (4–8 months old) of both sexes. Data were normalised to murine β-actin mRNA levels (p-value: 0.867) and β2m (p-value: 0.986) mRNA levels. The error bars represent the normalised standard deviation. Analyses were performed in three independent experiments using comparable groups of mice, within a similar age range (4–8 months) and with balanced sex distribution ($DH^{\Delta 4bp}$: F:6, M:3; $DH^{\Delta 4bp}$ *Ccnd3*-/-: F:5, M:4).

Figure S2

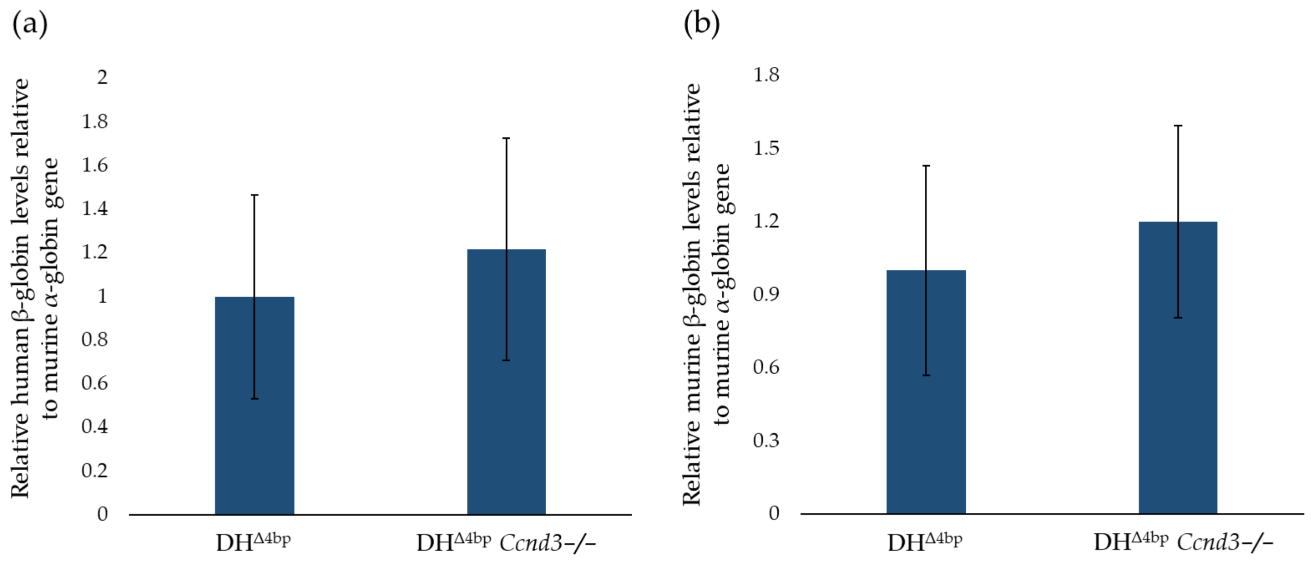

Figure S2. Relative expression levels of mutant (a) human and (b) murine β-globin transcript in bone marrow cells from $DH^{\Delta 4bp}$ and $DH^{\Delta 4bp}$ *Ccnd3*-/- adult mice, obtained by qPCR assay. mRNA expression data were normalised to mouse α-globin ($p = 0.639$ and 0.469). Analyses were performed in three independent experiments using comparable groups of mice, within a similar age range (4–8 months) and with balanced sex distribution ($DH^{\Delta 4bp}$: F:6, M:3; $DH^{\Delta 4bp}$ *Ccnd3*-/-: F:5, M:4). The error bars represent the normalised standard deviation.

Figure S3

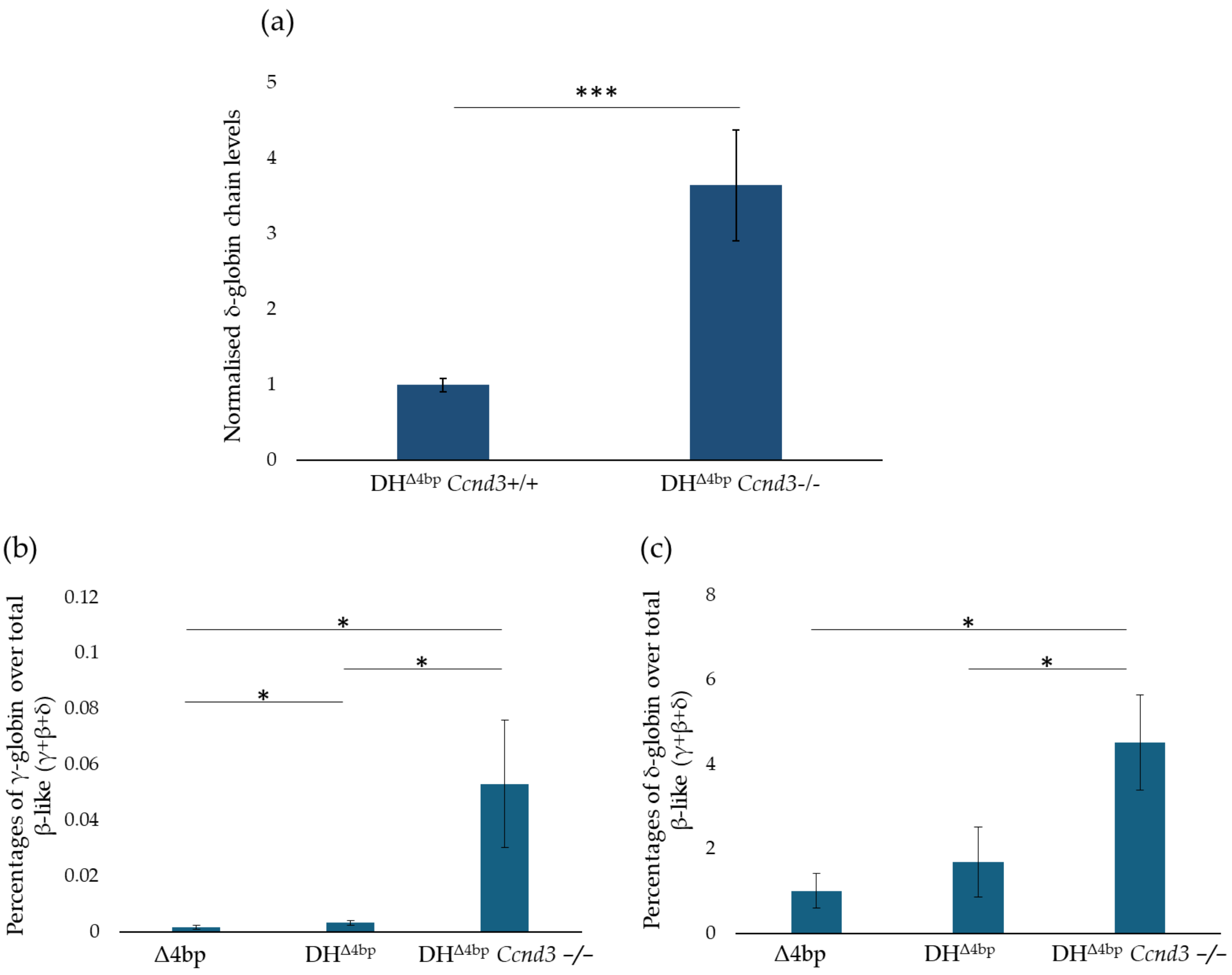

Figure S3. (a) Densitometric analysis of δ-globin bands from the Western blot shown in Figure 3e. Band intensities in DHΔ4bp Ccnd3-/- mice are expressed relative to the DHΔ4bp control and reported as relative densitometric units. Data represent the mean of three independent experiments; error bars indicate the normalised standard deviation ($p = 0.0009$). Analysis was performed using ImageJ software. (b) Levels of expression γ-globin and (c) δ-globin transcript in bone marrow cells from Δ4bp, DH$^{\Delta 4bp}$ and DH$^{\Delta 4bp}$ *Ccnd3-/-* adult mice obtained by qPCR assay. mRNA expression data were normalised to mouse α-globin. Expression levels of γ- and δ-globin are presented as a percentage relative to total beta-like globins (human γ + human δ + murine β). The error bars represent the standard deviation. (p-value: * < 0.05). Analyses were performed in three independent experiments using comparable groups of mice, within a similar age range (4–8 months) and with balanced sex distribution (Δ4bp: F:5, M:4; DH$^{\Delta 4bp}$: F:6, M:3; DH$^{\Delta 4bp}$ *Ccnd3-/-*: F:5, M:4).

Figure S4

a b

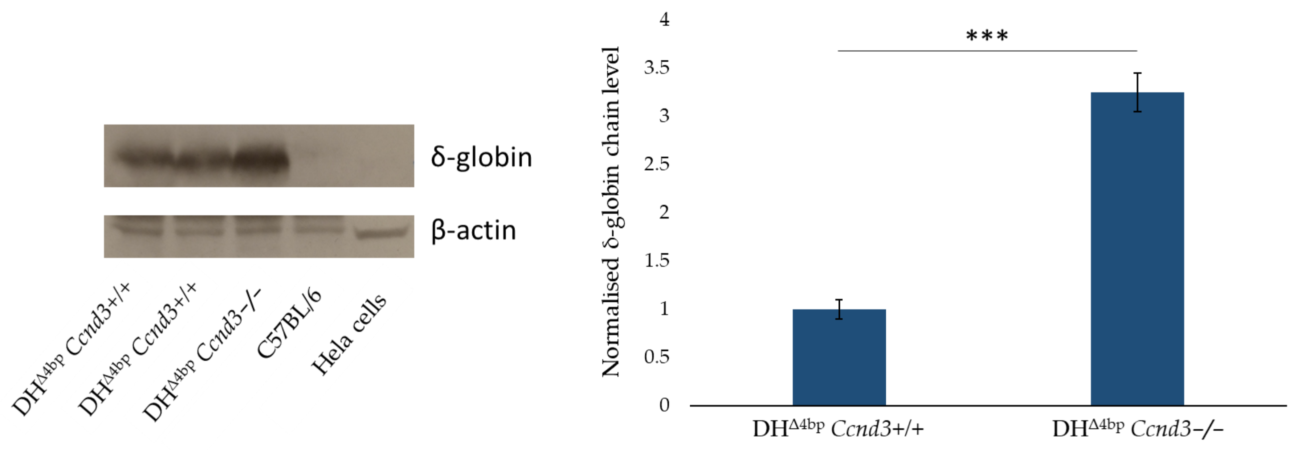

Figure S4. (a) Representative western blot analysis of δ-globin chain expression in peripheral blood from adult DH$^{\Delta 4bp}$ and DH$^{\Delta 4bp}$ *Ccnd3-/-* mice. Lane 1–2: DH$^{\Delta 4bp}$ mice; lane 3: DH$^{\Delta 4bp}$ *Ccnd3-/-* mouse; lane 4: C57BL/6 wild-type peripheral blood (negative control); lane 5: K562 cells (negative control). β-actin was used as endogenous control. The blot shown is representative of three independent experiments. Animals were 4–8 months old and of comparable sex. (b) Densitometric analysis performed using ImageJ software. The graph shows δ-globin band intensities of DH$^{\Delta 4bp}$ *Ccnd3-/-* mice relative to the mean of the two DH$^{\Delta 4bp}$ controls, expressed as relative densitometric units. Error bars represent the normalised standard deviation between biological replicates (p-value: 0.00045)

Figure S5

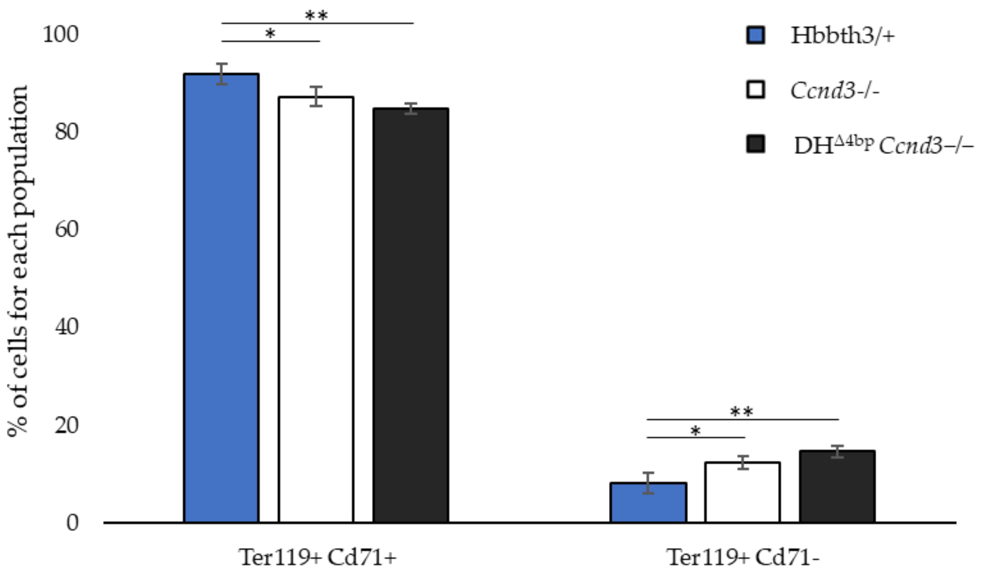

Figure S5. Flow cytometry analysis of erythropoiesis in Hbbth3/+, *Ccnd3*-/- and DH$^{\Delta4bp}$ *Ccnd3*-/- bone marrow freshly isolated cells. Analysis was conducted considering Ter119 and Cd71 markers levels of expression. At least three mice from each group within a similar age range (4–8 months) and with balanced sex distribution (Hbbth3/+: F:2, M:1; *Ccnd3*-/-: F:1, M:2; DH$^{\Delta4bp}$ *Ccnd3*-/-: F:2, M:1) were analysed. The error bars represent the standard deviation. (p-value: * < 0.05; **< 0.01).

Figure S6

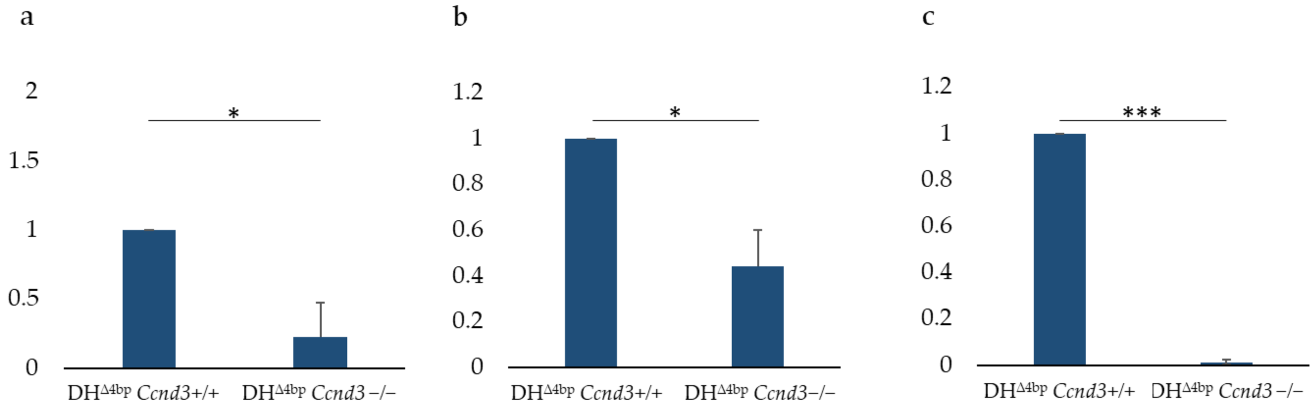

Figure S6. Frequency of erythrocytes with thalassemia-associated morphological features in $DH^{\Delta 4bp}$ *Ccnd3-/-* mice, normalised to $DH^{\Delta 4bp}$ controls. (a) Target cells ($p = 0.043$), (b) Schistocytes ($p = 0.013$), (c) Burr cells ($p = 0.00076$). Data were obtained from 5 animals per group ($DH^{\Delta 4bp}$: 3 females, 2 males; $DH^{\Delta 4bp}$ *Ccnd3-/-*: 2 females, 3 males; age range 4–8 months). The error bars represent the normalised standard deviation.

Figure S7

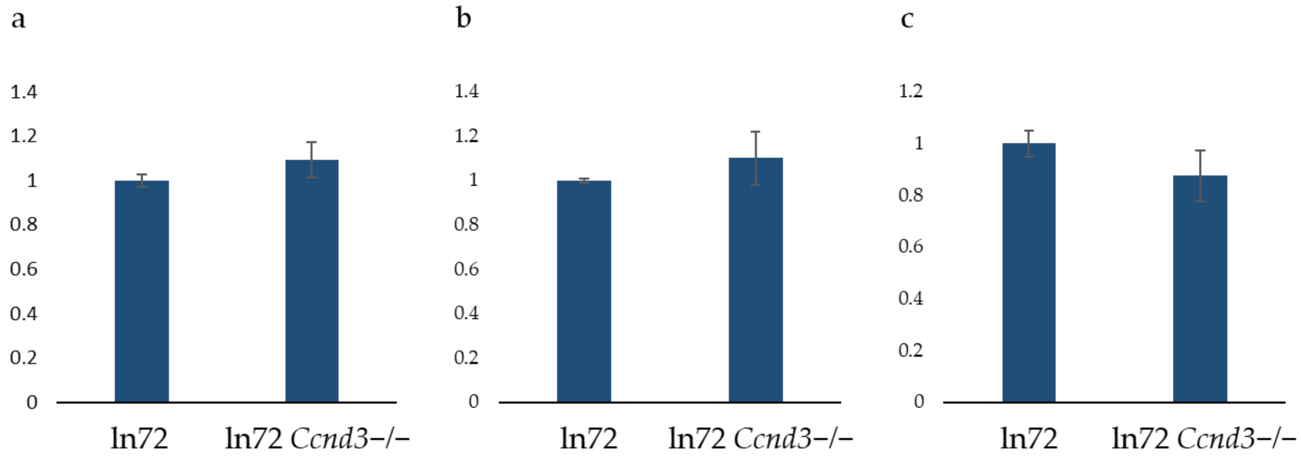

Figure S7. Relative expression levels of (a) *Fasl* (pvalue: 0.74), (b) *Bcl-xl* (pvalue: 0.38) and (c) *Epor* (pvalue: 0.24) transcripts in bone marrow cells from ln72 and ln72 *Ccnd3-/-* adult mice, obtained by qPCR assay. mRNA expression data were normalised to mouse α-globin. Analyses were performed on 5 samples per group using comparable groups of mice with similar age range and sex distribution. The error bars represent the normalised standard deviation (pvalue)
